# Supplementary figures and images for: Stress granules are not present in Kras mutant cancers and do not control tumor growth
Source: EMBO Rep. 2024 Oct 10;25(11):7. doi: 10.1038/s44319-024-00284-6 (PMC11549491; doi:10.1038/s44319-024-00284-6)

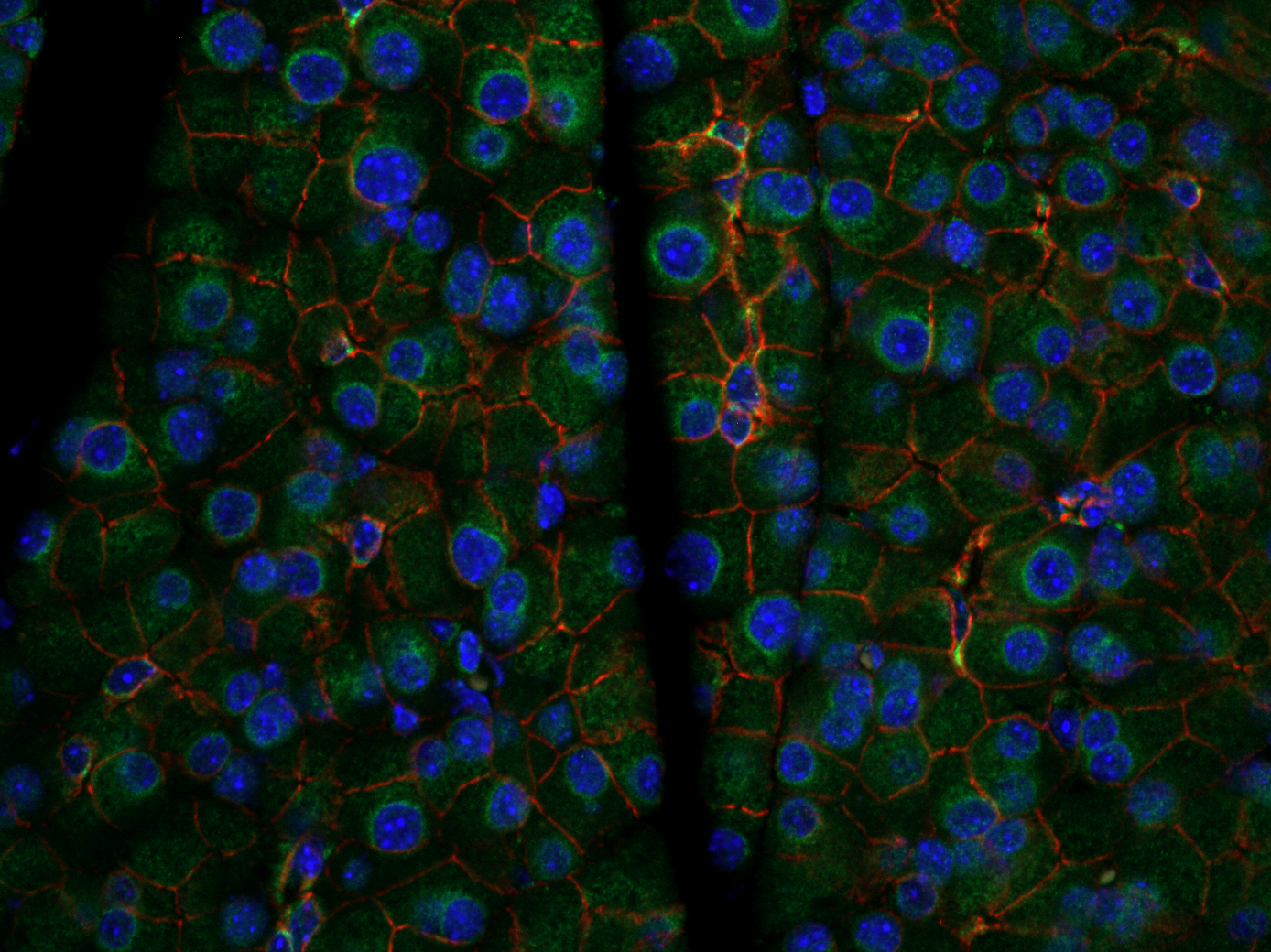

Supplement: Supplementary file 4 — Source data Fig. 1A [file 44319_2024_284_MOESM4_ESM.zip › Figure1A_SD/ElaK.tif]

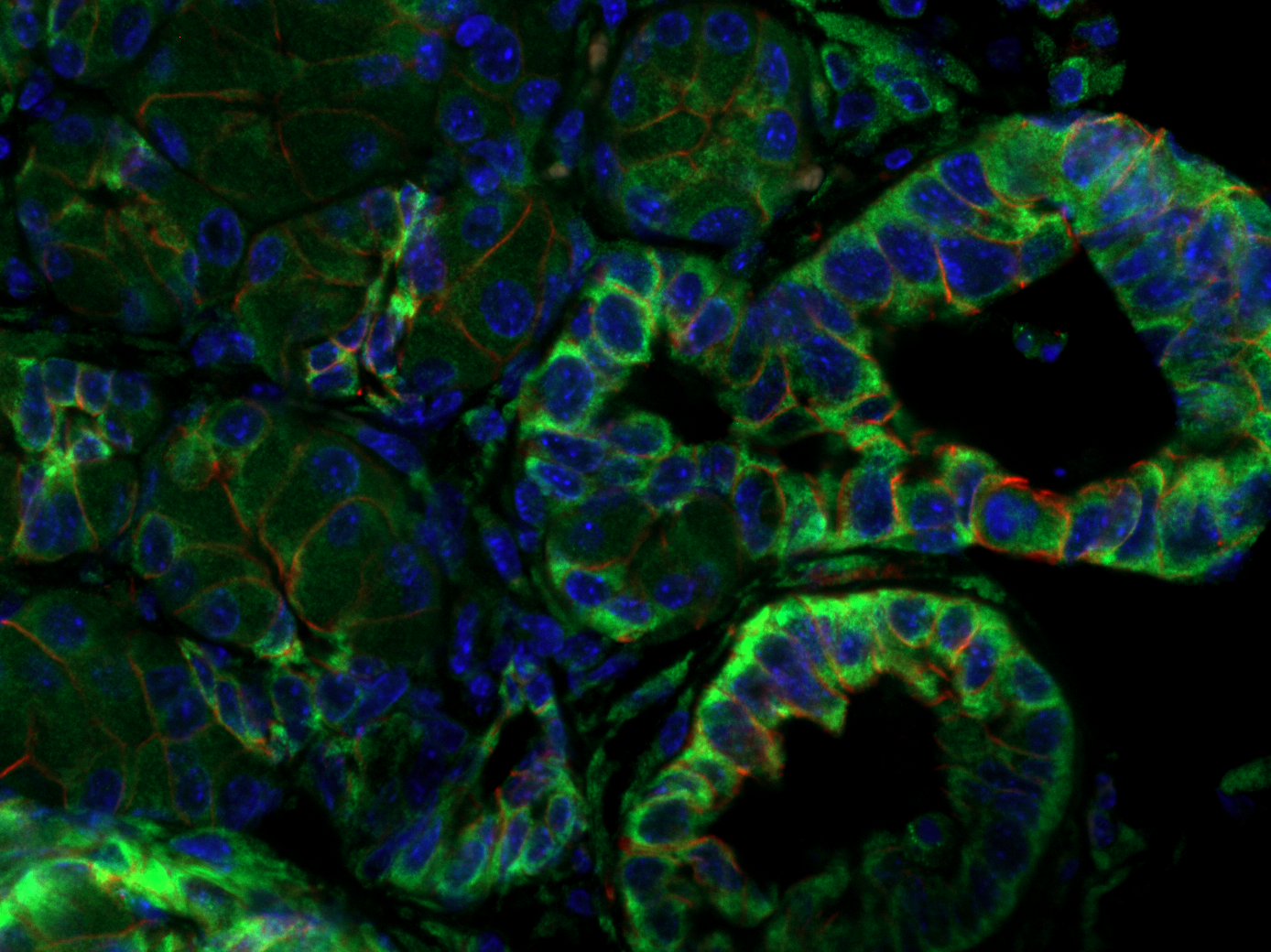

Supplement: Supplementary file 4 — Source data Fig. 1A [file 44319_2024_284_MOESM4_ESM.zip › Figure1A_SD/ElaK+cerulein.tif]

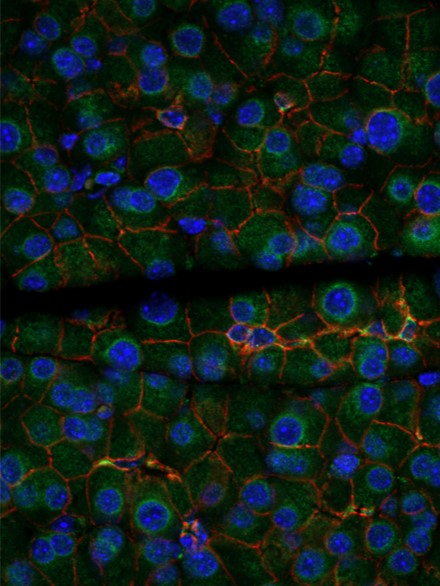

Supplement: Supplementary file 4 — Source data Fig. 1A [file 44319_2024_284_MOESM4_ESM.zip › Figure1A_SD/ElaK.jpg]

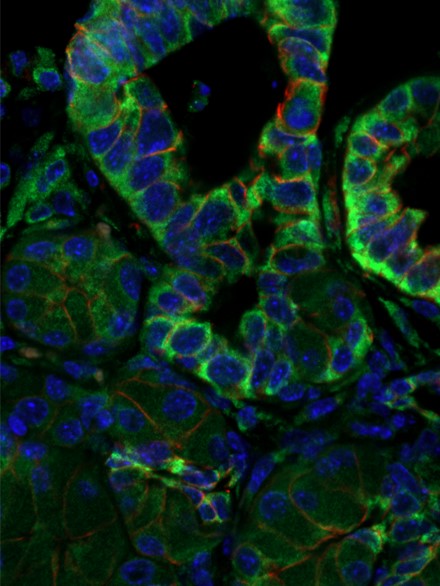

Supplement: Supplementary file 4 — Source data Fig. 1A [file 44319_2024_284_MOESM4_ESM.zip › Figure1A_SD/ElaK+cerulein.jpg]

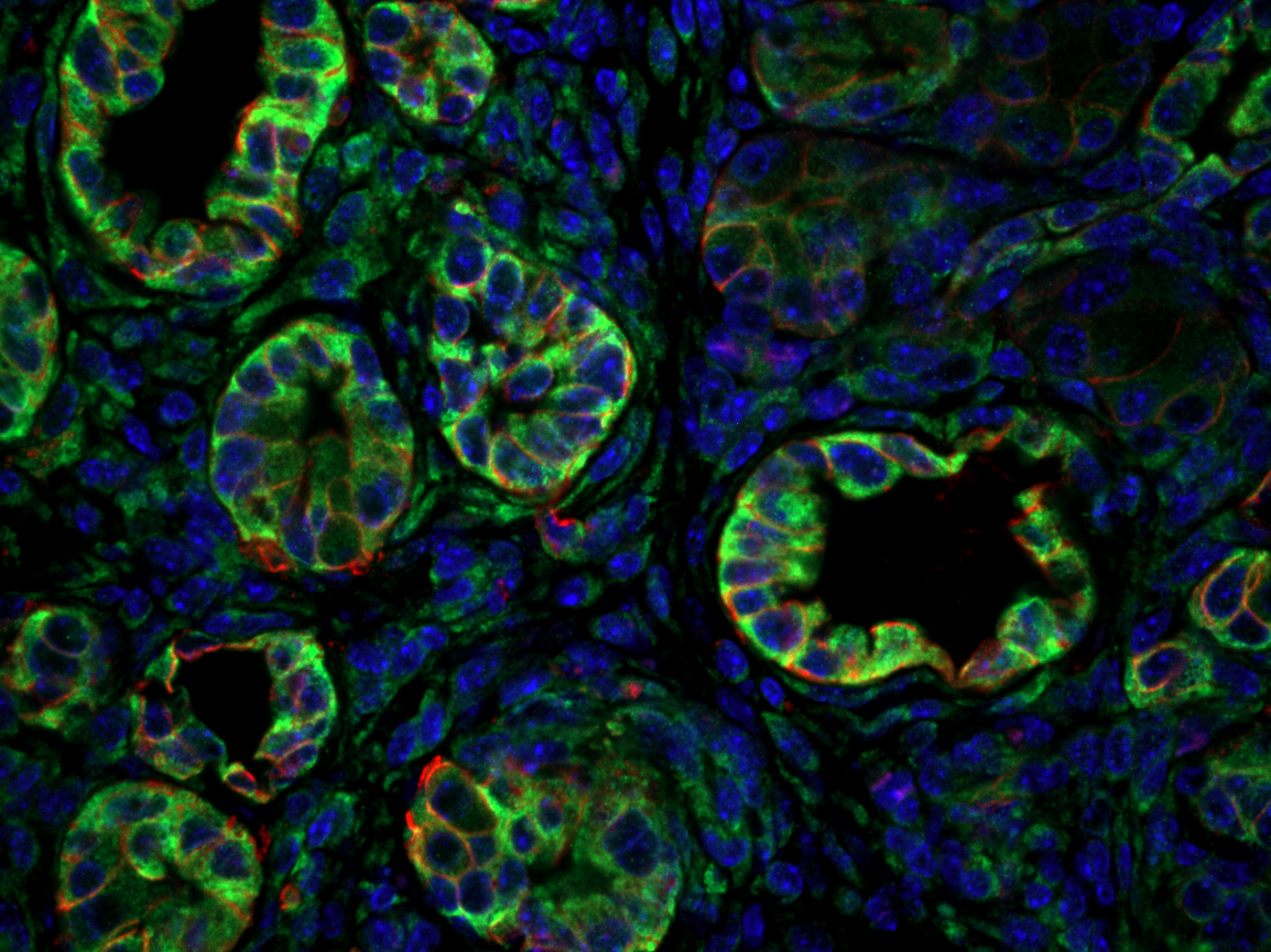

Supplement: Supplementary file 5 — Source data Fig. 1B [file 44319_2024_284_MOESM5_ESM.zip › 1B/ElaK+cerulein.tiff]

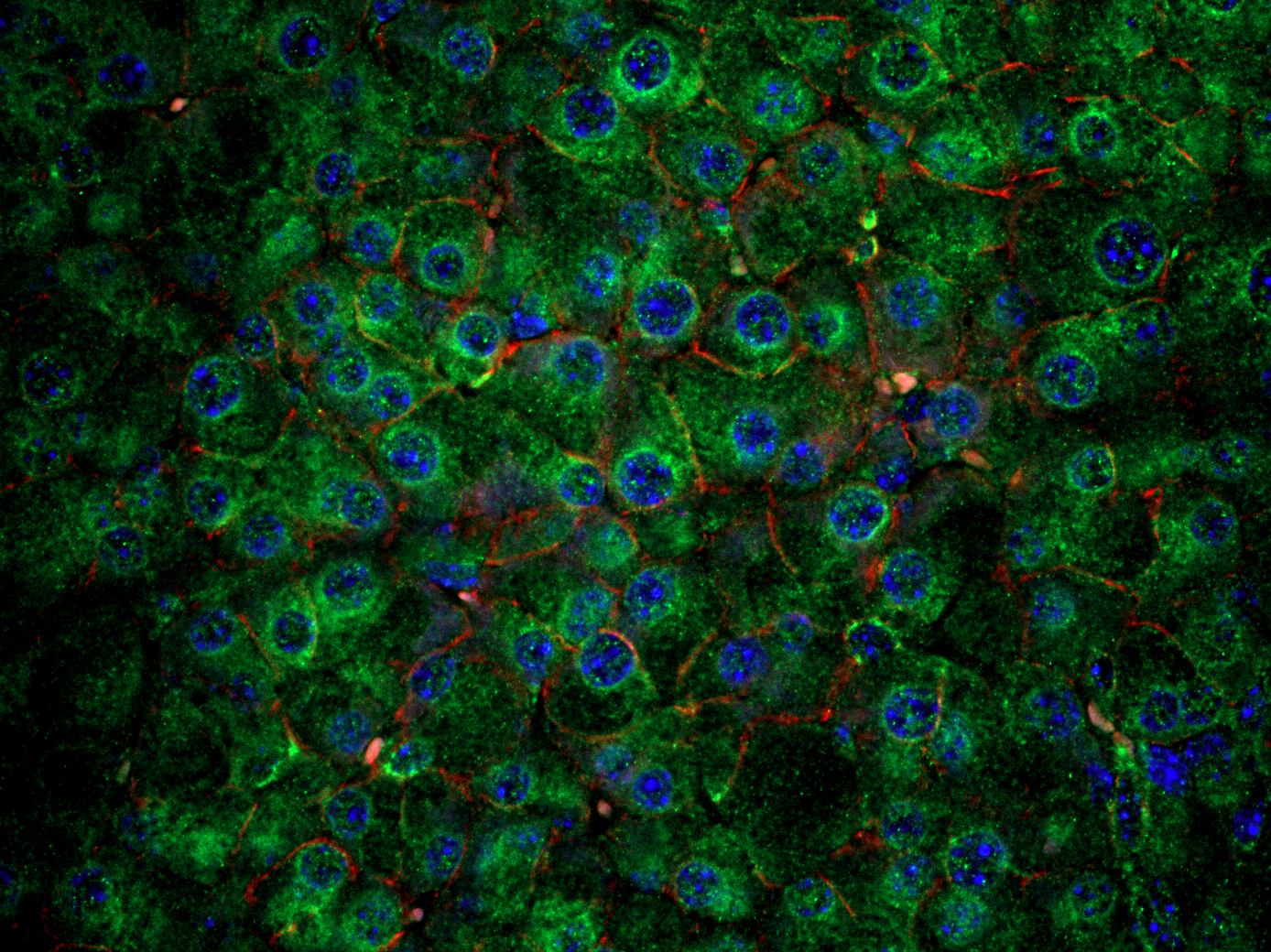

Supplement: Supplementary file 5 — Source data Fig. 1B [file 44319_2024_284_MOESM5_ESM.zip › 1B/ElaK.tif]

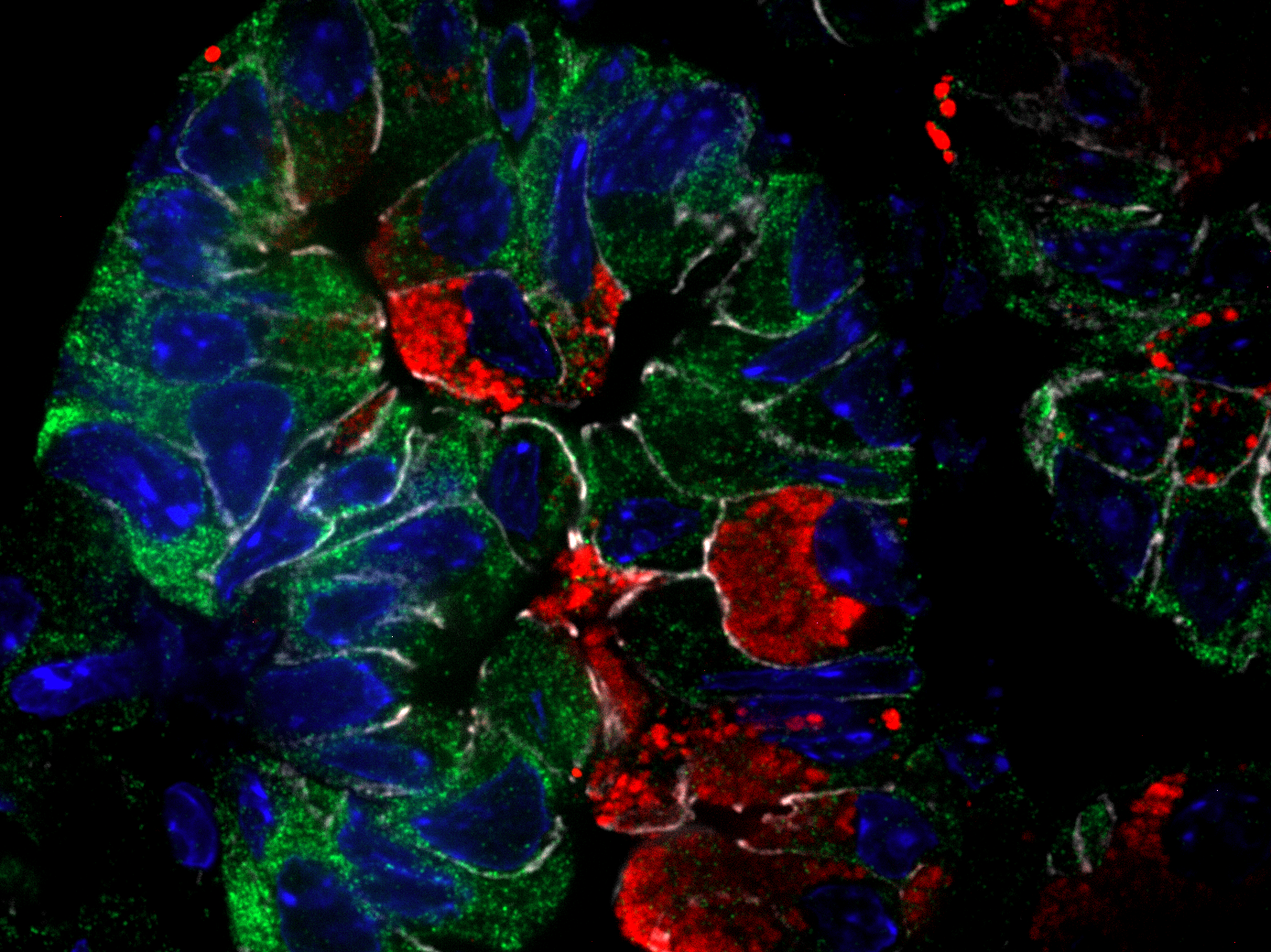

Supplement: Supplementary file 6 — Source data Fig. 1C_F [file 44319_2024_284_MOESM6_ESM.zip › SG_EMBOR_SourceData_Fig1C_F/1F/Control.tif]

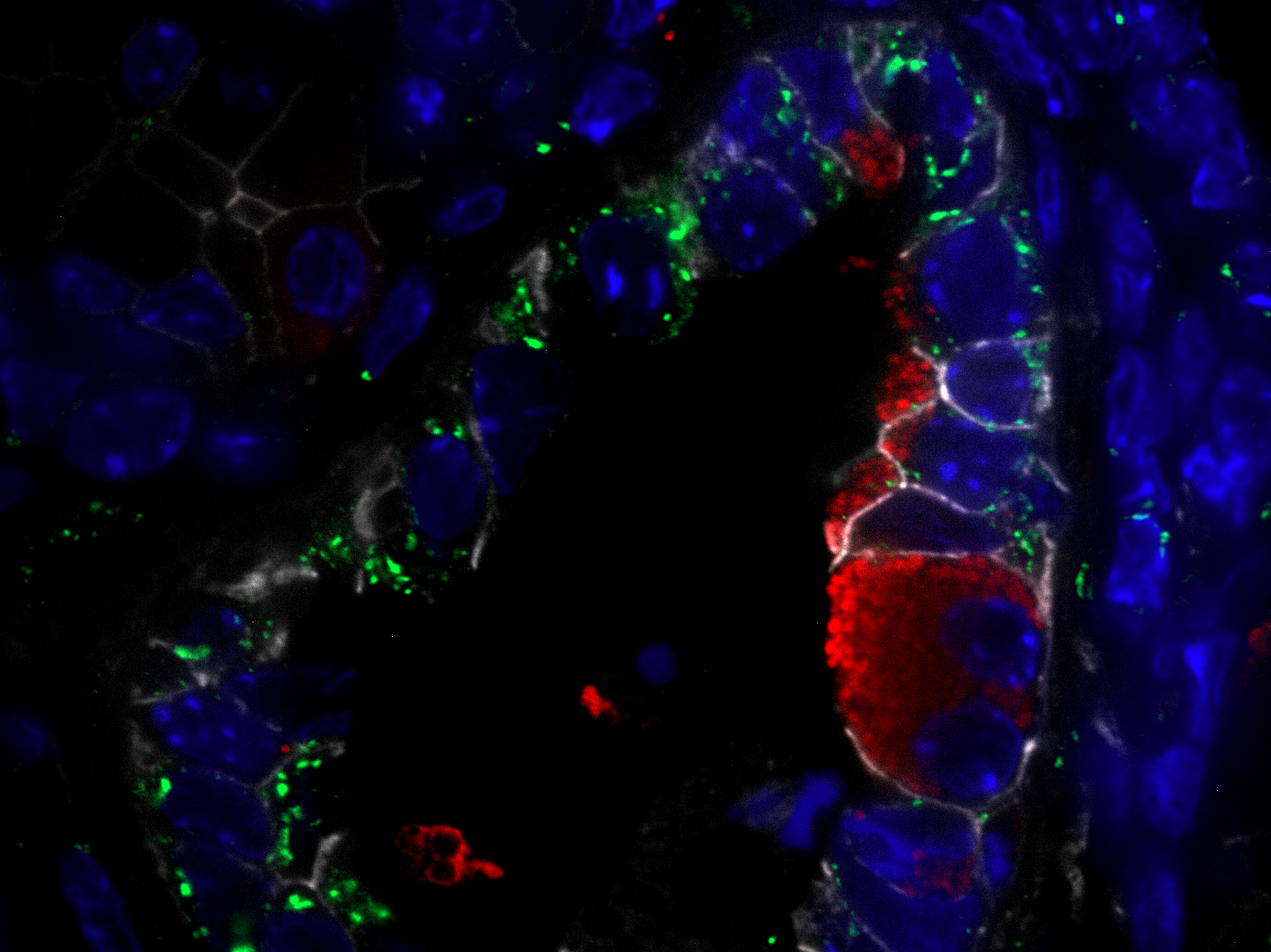

Supplement: Supplementary file 6 — Source data Fig. 1C_F [file 44319_2024_284_MOESM6_ESM.zip › SG_EMBOR_SourceData_Fig1C_F/1F/Heat_Shock.tif]

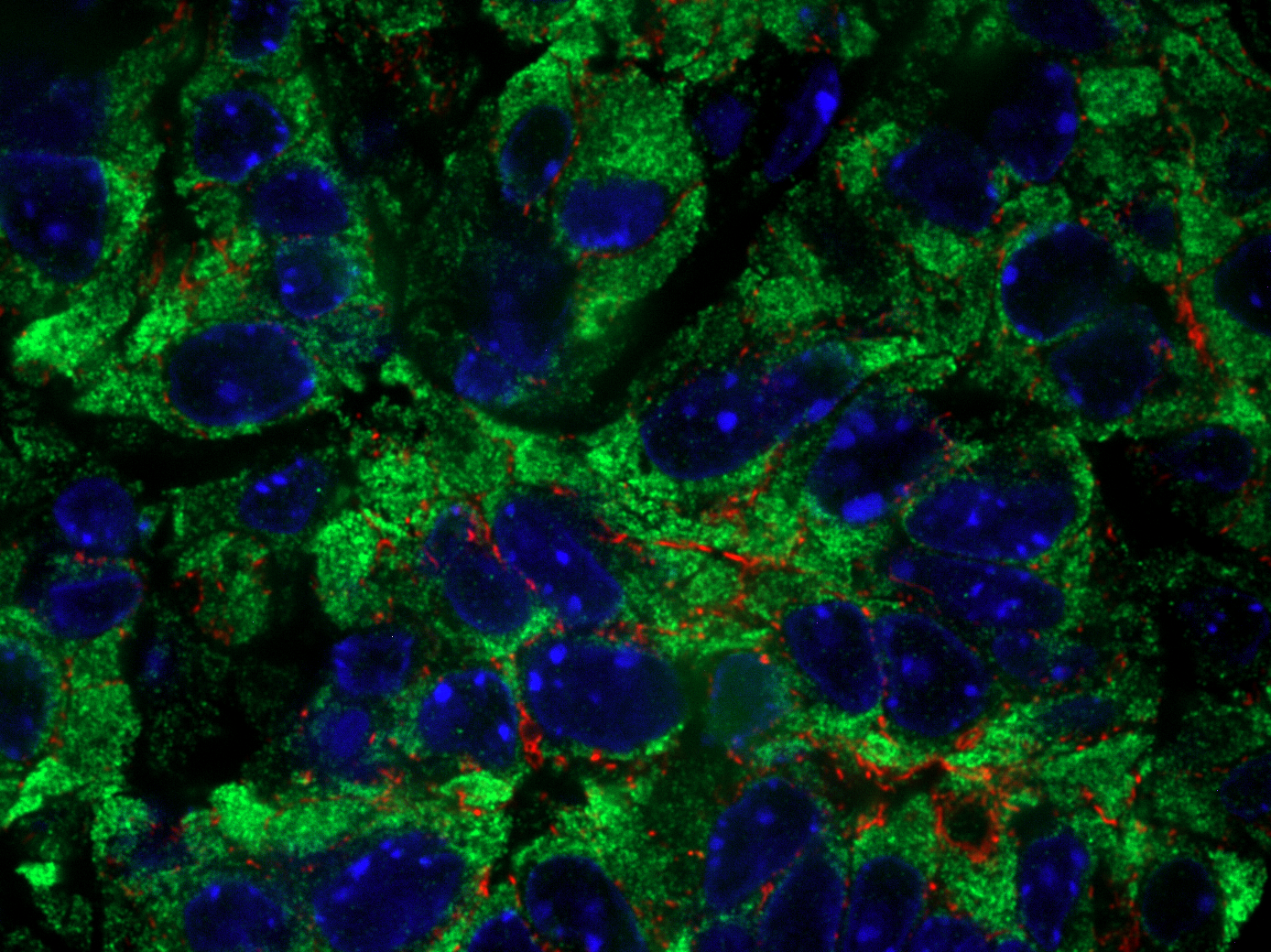

Supplement: Supplementary file 6 — Source data Fig. 1C_F [file 44319_2024_284_MOESM6_ESM.zip › SG_EMBOR_SourceData_Fig1C_F/1E/Orthotopic_graft.tif]

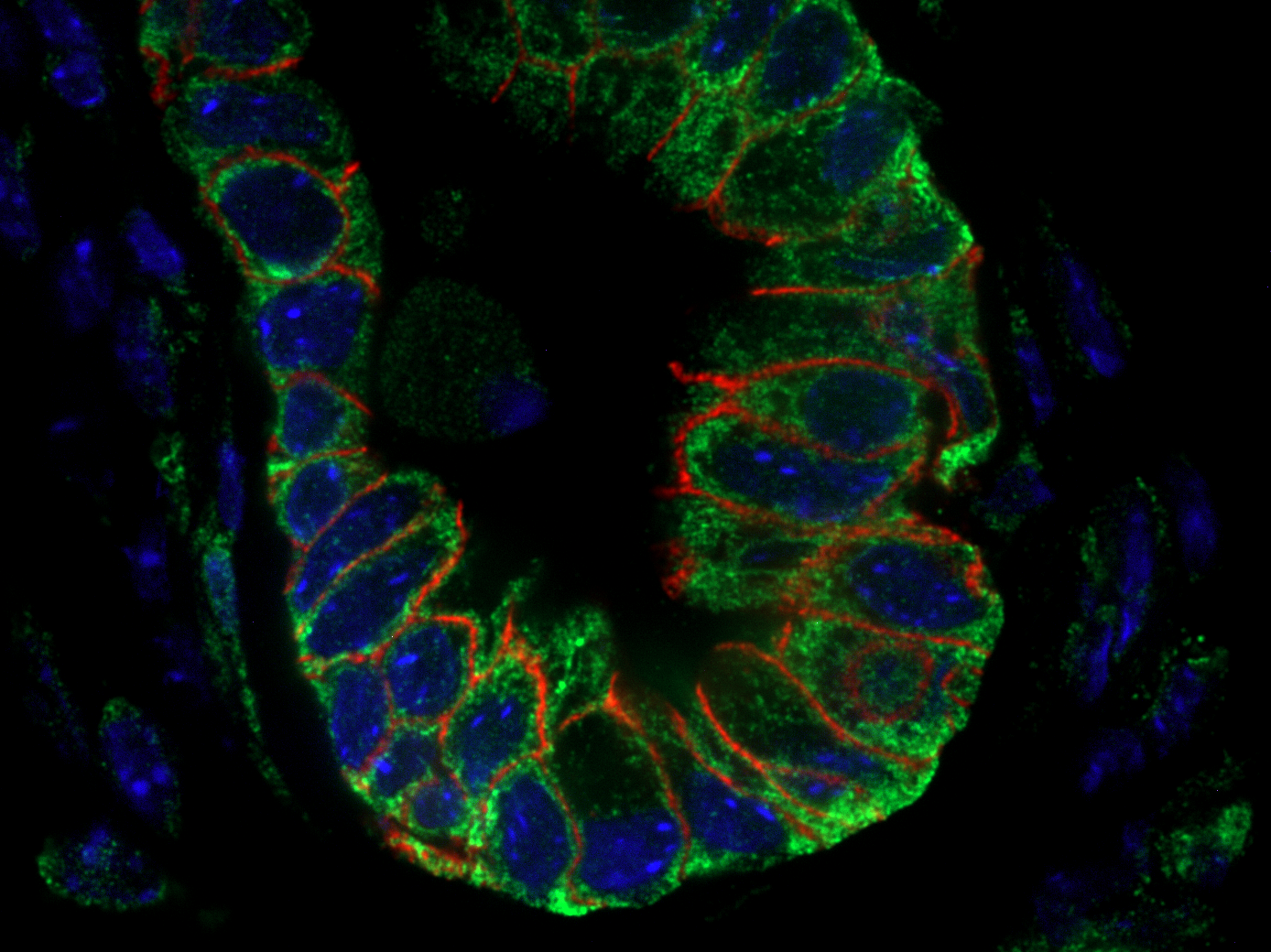

Supplement: Supplementary file 6 — Source data Fig. 1C_F [file 44319_2024_284_MOESM6_ESM.zip › SG_EMBOR_SourceData_Fig1C_F/1E/ElaKP_mice.tif]

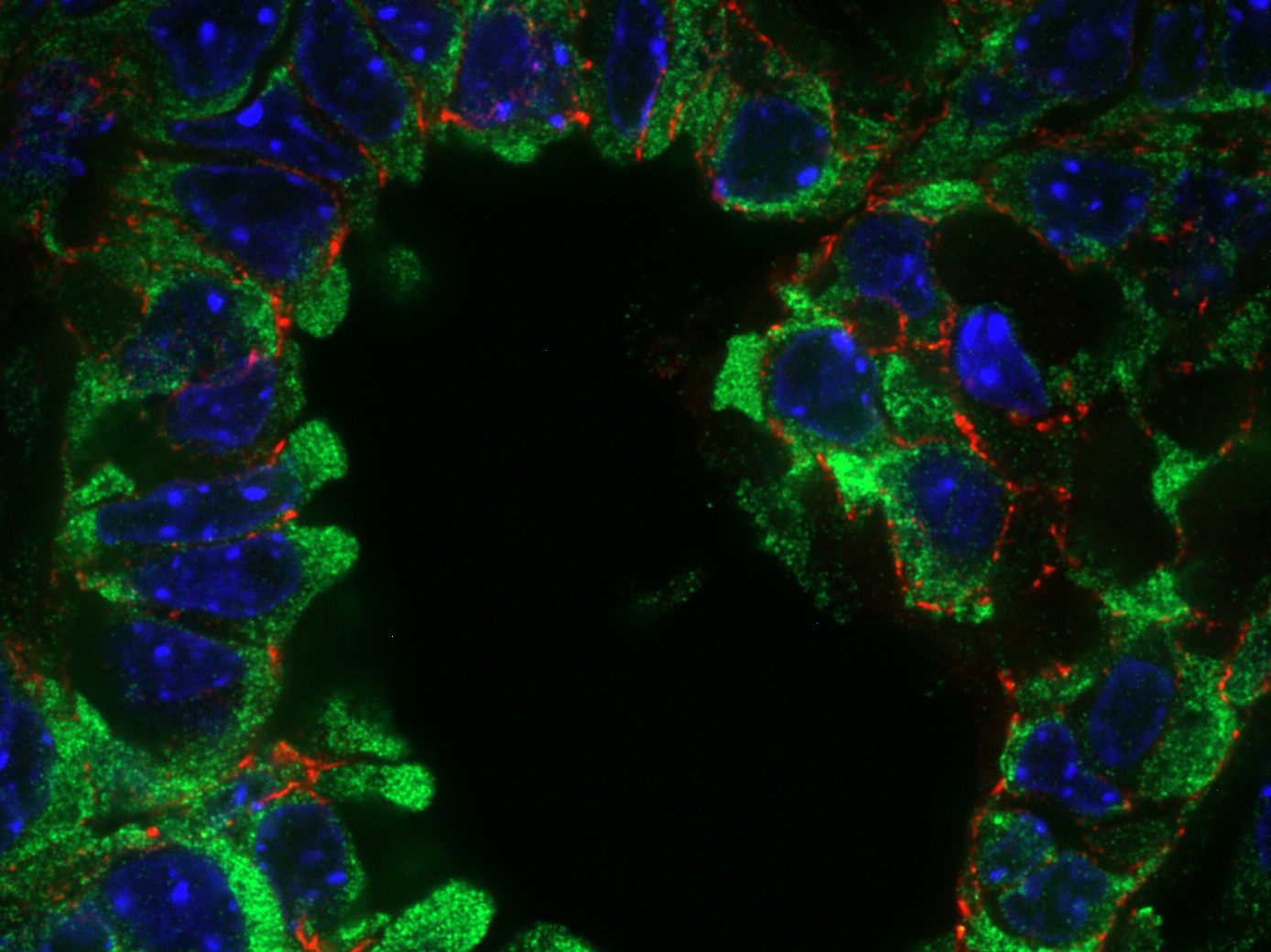

Supplement: Supplementary file 6 — Source data Fig. 1C_F [file 44319_2024_284_MOESM6_ESM.zip › SG_EMBOR_SourceData_Fig1C_F/1D/Orthotopic_graft.tif]

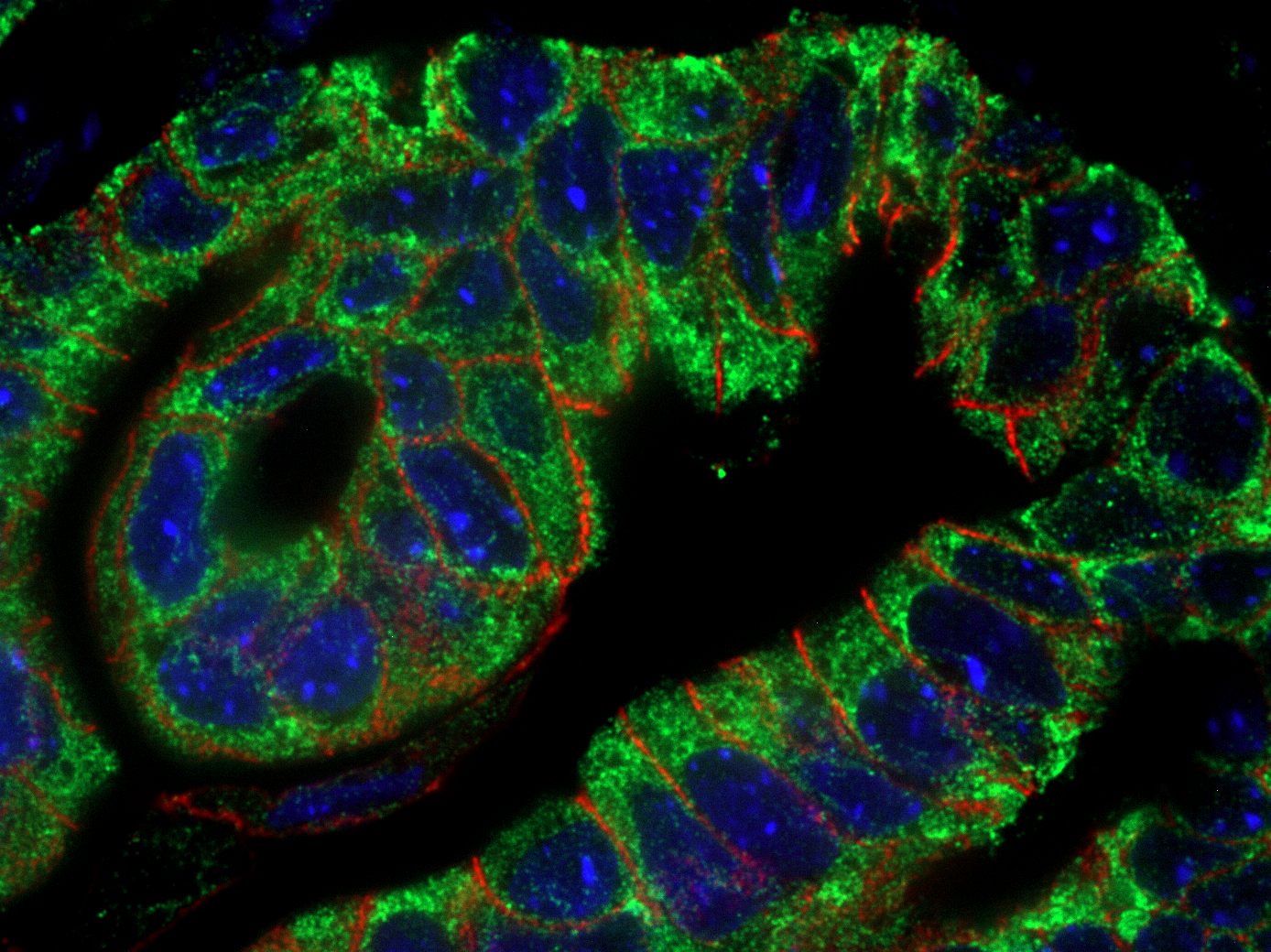

Supplement: Supplementary file 6 — Source data Fig. 1C_F [file 44319_2024_284_MOESM6_ESM.zip › SG_EMBOR_SourceData_Fig1C_F/1D/ElaKP_mice.tif]

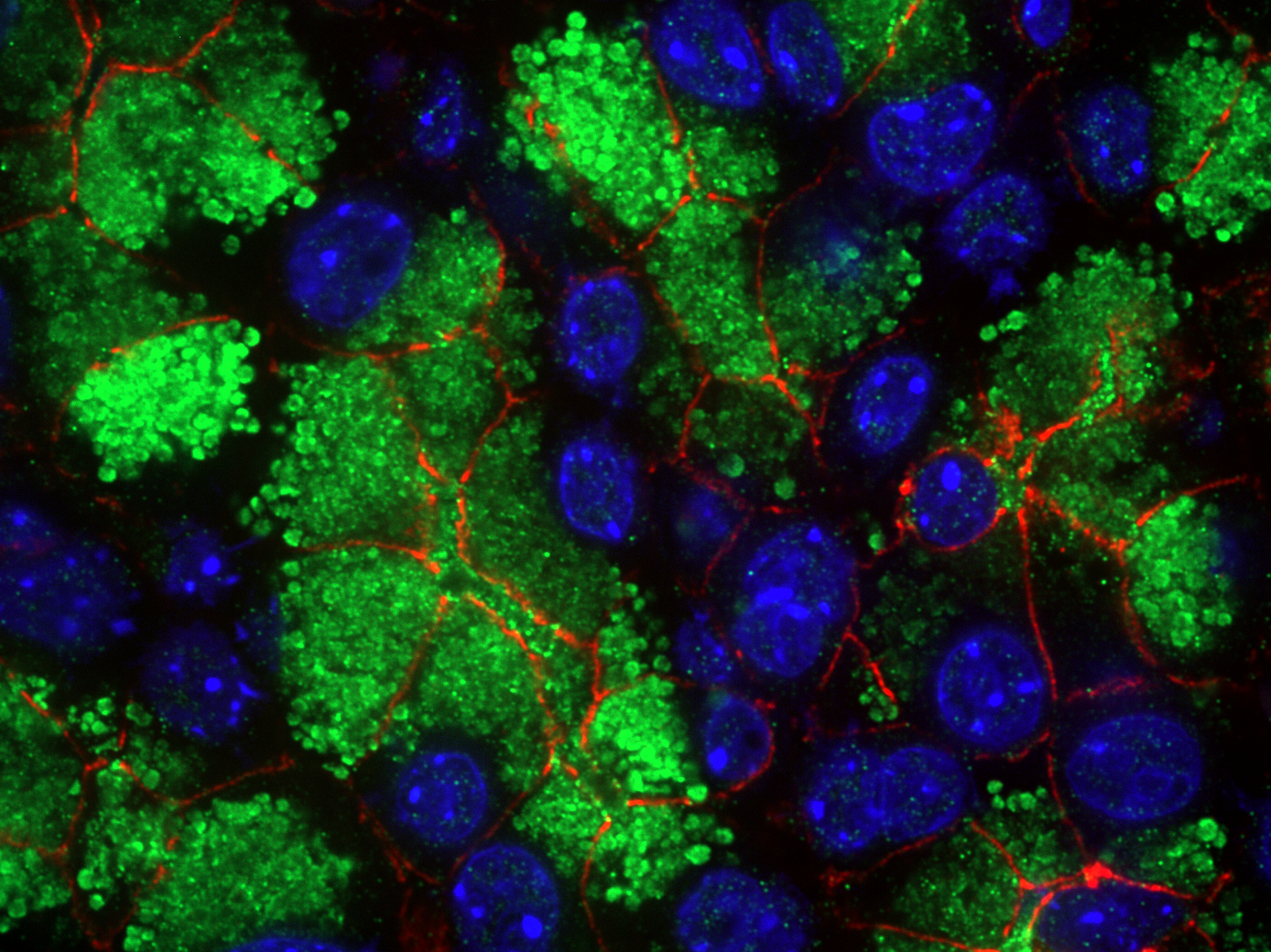

Supplement: Supplementary file 6 — Source data Fig. 1C_F [file 44319_2024_284_MOESM6_ESM.zip › SG_EMBOR_SourceData_Fig1C_F/1C/ElaK.tif]

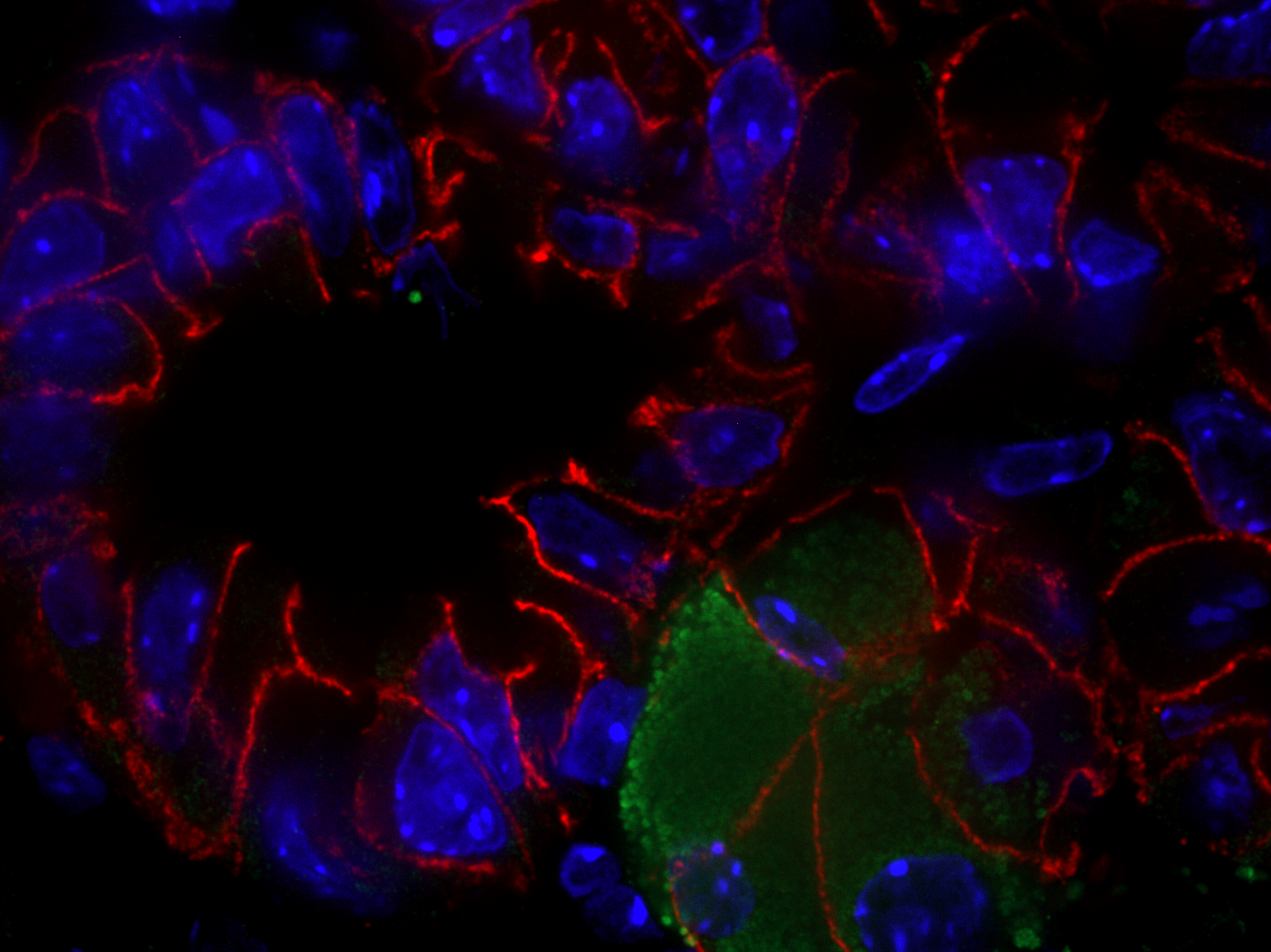

Supplement: Supplementary file 6 — Source data Fig. 1C_F [file 44319_2024_284_MOESM6_ESM.zip › SG_EMBOR_SourceData_Fig1C_F/1C/ElaK+cerulein.tif]

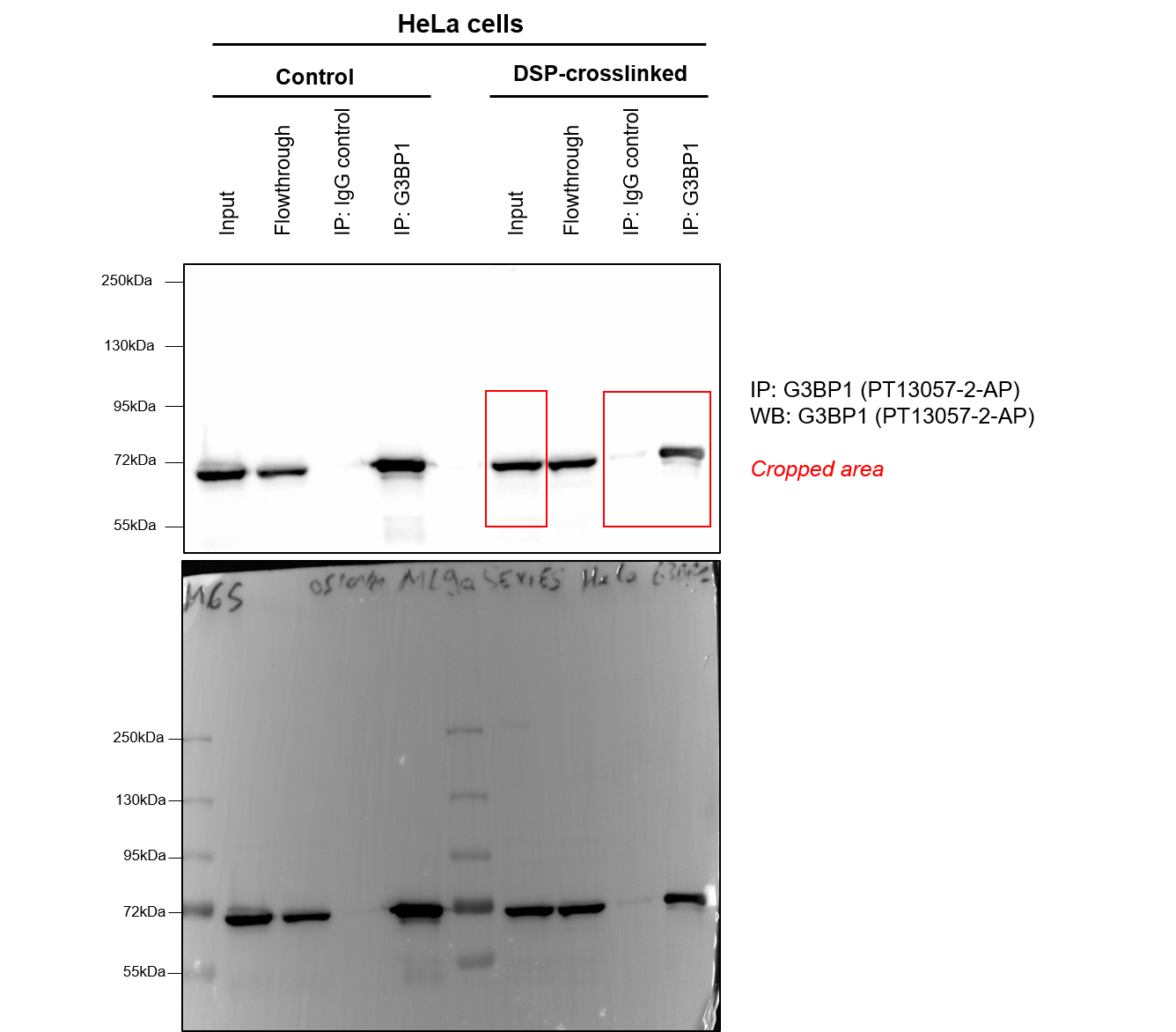

Supplement: Supplementary file 7 — Source data Fig. 2 [file 44319_2024_284_MOESM7_ESM.zip › Figure 2A_SD/2A Western Blot.tif]

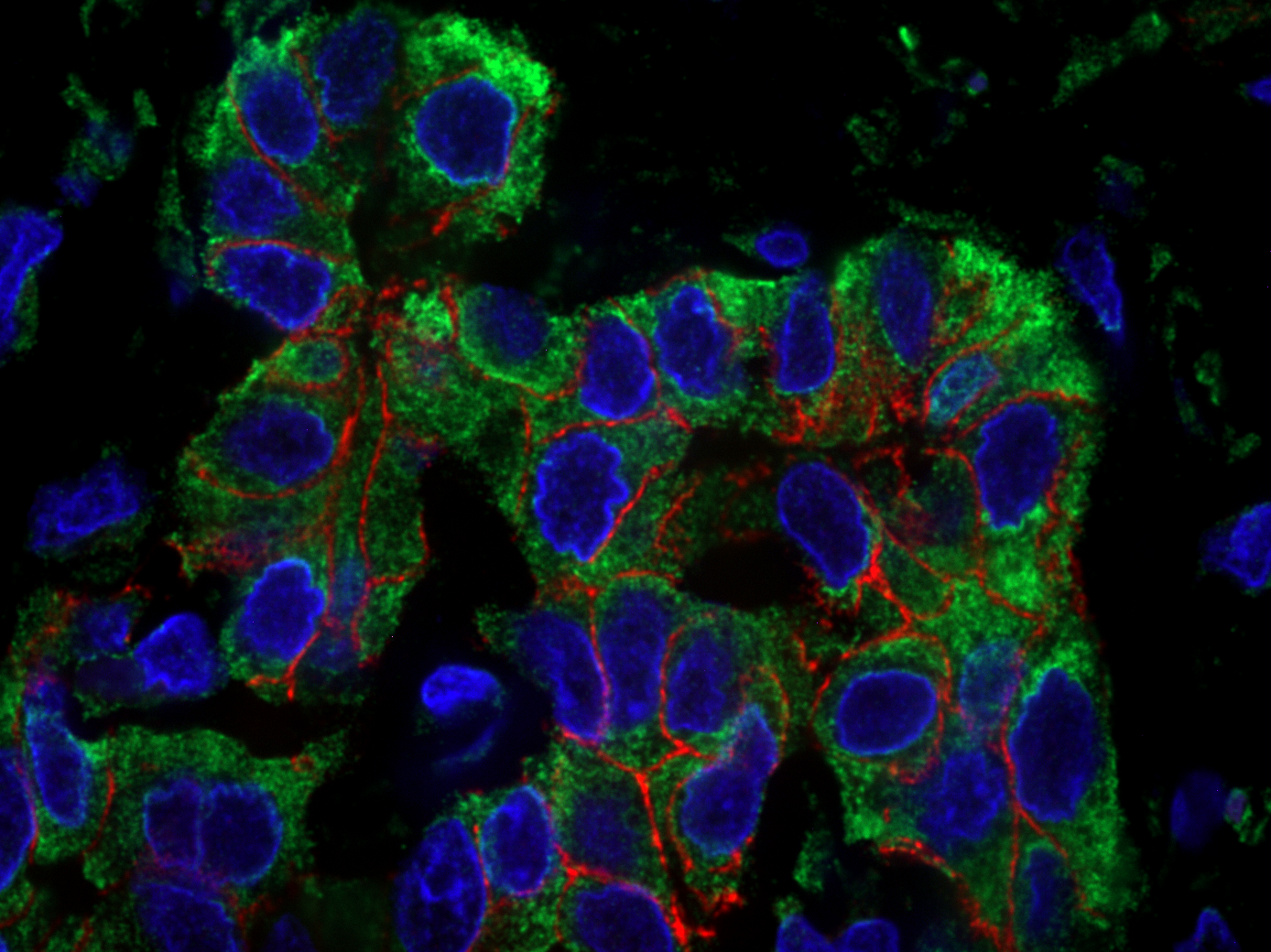

Supplement: Supplementary file 9 — Source data Fig. 5 [file 44319_2024_284_MOESM9_ESM.zip › Figure 5_SD/5A/Image1.tif]

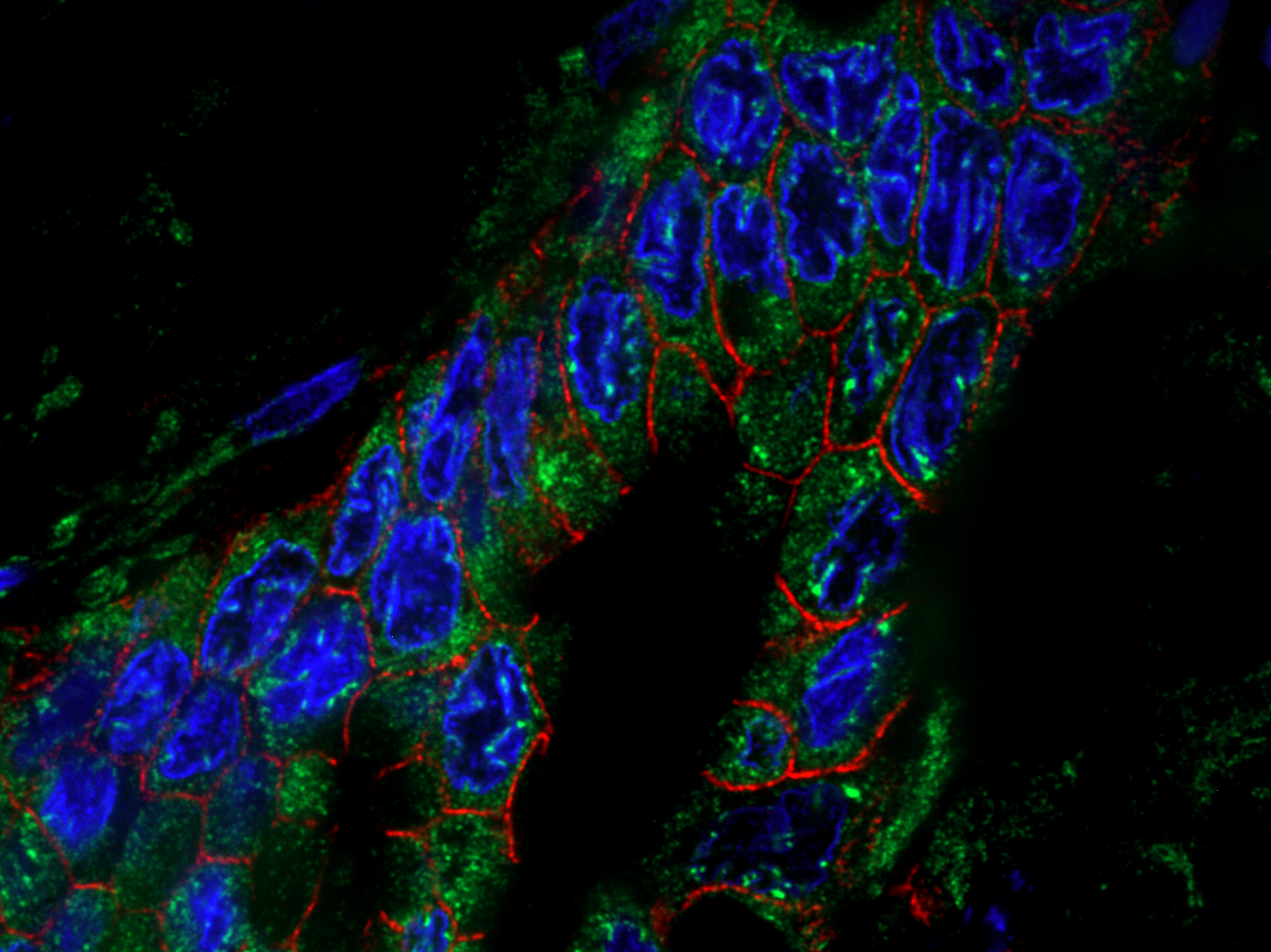

Supplement: Supplementary file 9 — Source data Fig. 5 [file 44319_2024_284_MOESM9_ESM.zip › Figure 5_SD/5A/Image3.tif]

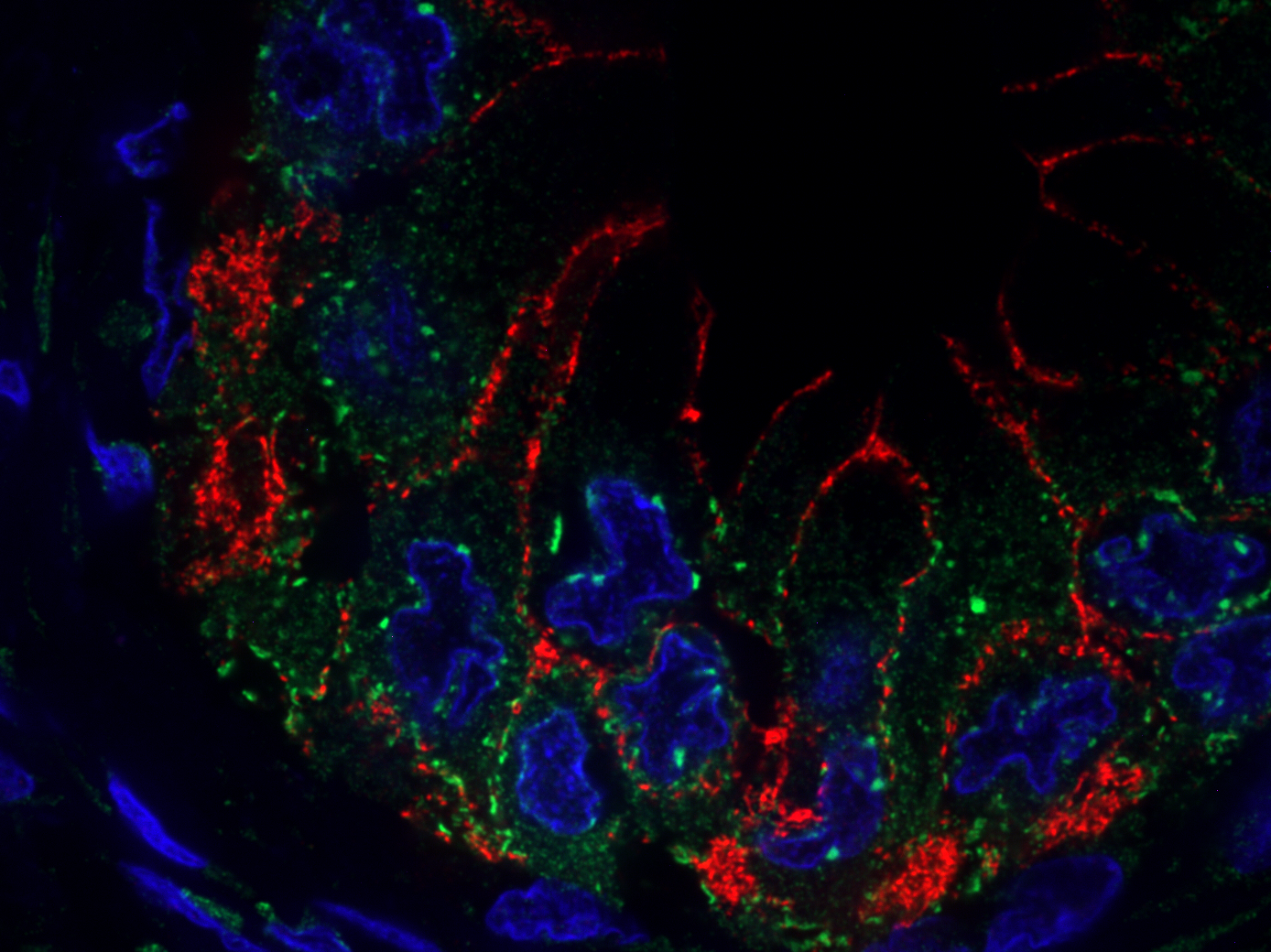

Supplement: Supplementary file 9 — Source data Fig. 5 [file 44319_2024_284_MOESM9_ESM.zip › Figure 5_SD/5A/Image2.tif]

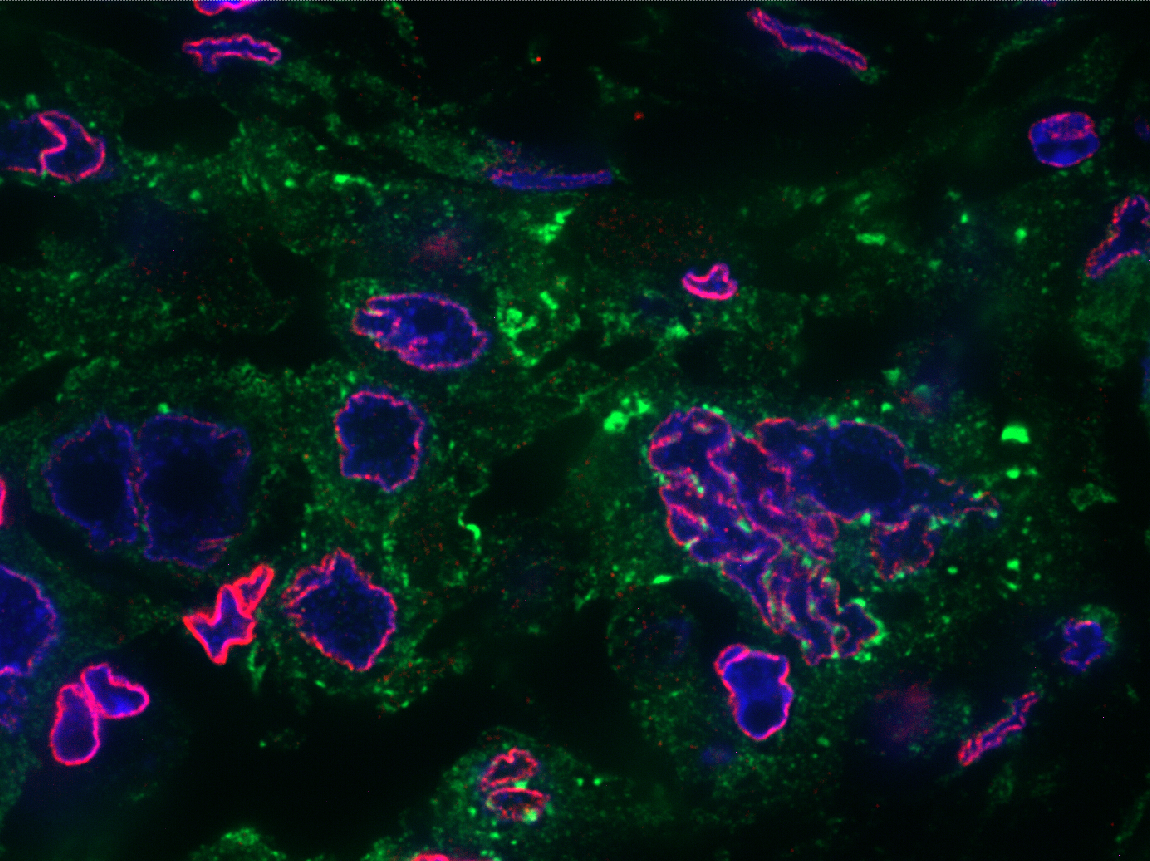

Supplement: Supplementary file 9 — Source data Fig. 5 [file 44319_2024_284_MOESM9_ESM.zip › Figure 5_SD/5B/Image1.tif]

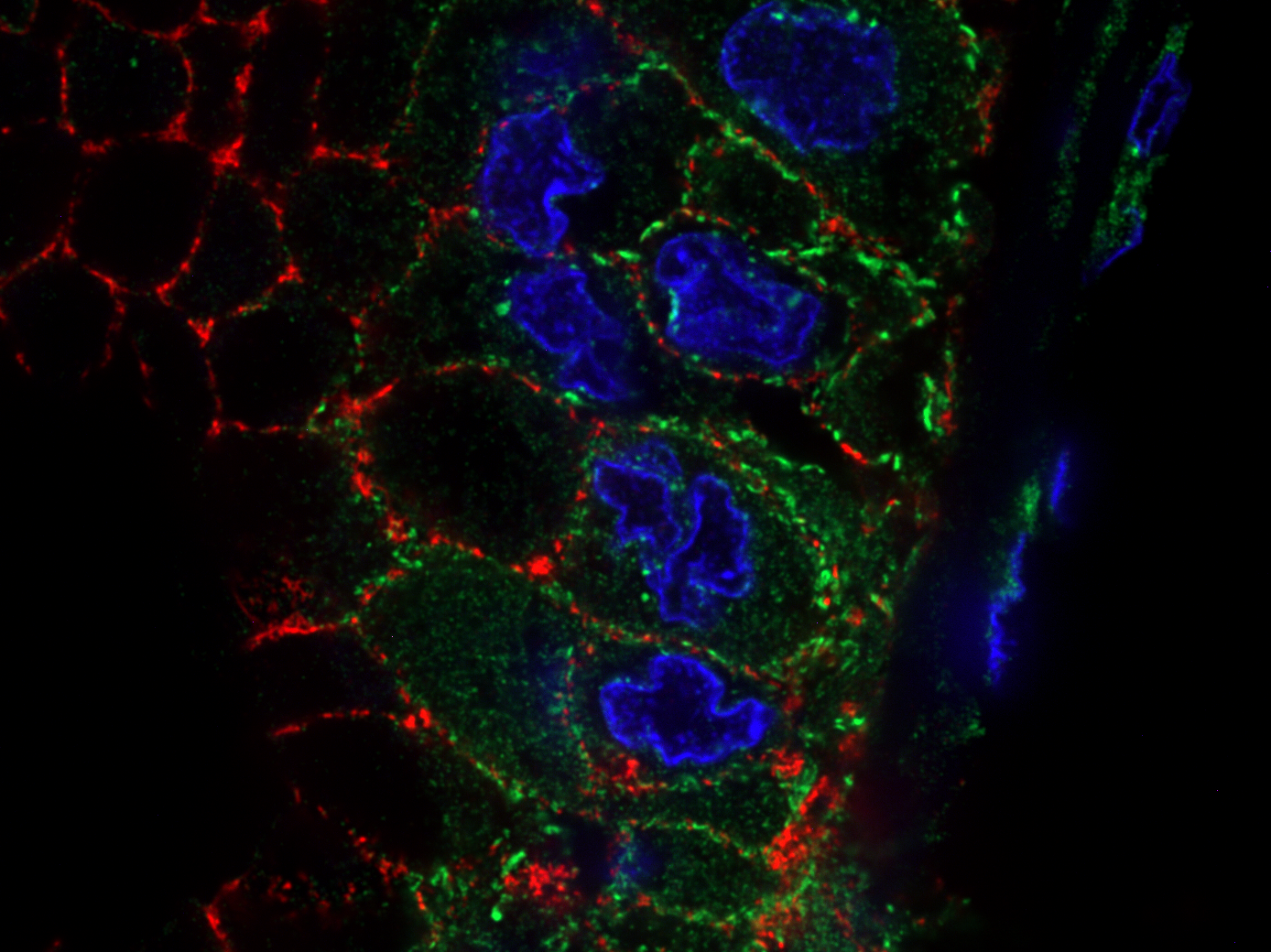

Supplement: Supplementary file 9 — Source data Fig. 5 [file 44319_2024_284_MOESM9_ESM.zip › Figure 5_SD/5B/Image2.tif]
