# Supplementary material for: Stress granules are not present in Kras mutant cancers and do not control tumor growth
Source: EMBO Rep. 2024 Oct 10;25(11):7. doi: 10.1038/s44319-024-00284-6 (PMC11549491; doi:10.1038/s44319-024-00284-6)
Supplement: Supplementary file 7 — Source data Fig. 2 [file 44319_2024_284_MOESM7_ESM.zip › Figure 2A_SD/2A Western Blot.pptx]

## Slide 1
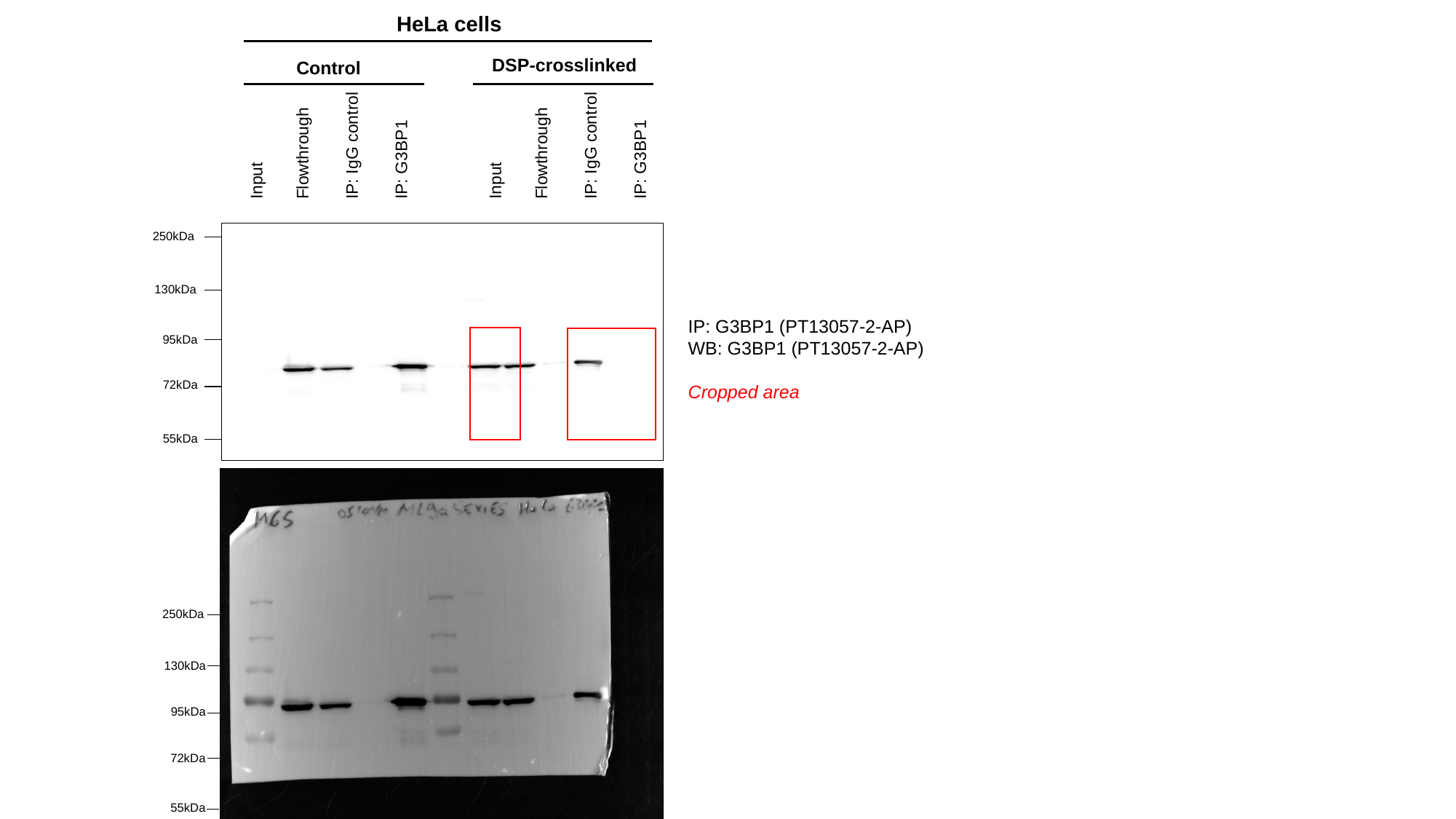

HeLa cells
DSP-crosslinked
Control
Flowthrough
IP: IgG control
IP: G3BP1
Input
Flowthrough
IP: IgG control
IP: G3BP1
Input
250kDa
130kDa
95kDa
72kDa
55kDa
IP: G3BP1 (PT13057-2-AP)
WB: G3BP1 (PT13057-2-AP)
Cropped area
250kDa
130kDa
95kDa
72kDa
55kDa
