# Supplementary material for: Stress granules are not present in Kras mutant cancers and do not control tumor growth
Source: EMBO Rep. 2024 Oct 10;25(11):7. doi: 10.1038/s44319-024-00284-6 (PMC11549491; doi:10.1038/s44319-024-00284-6)
Supplement: Supplementary file 10 — Expanded View Figures [file 44319_2024_284_MOESM10_ESM.pdf]

Expanded View Figures

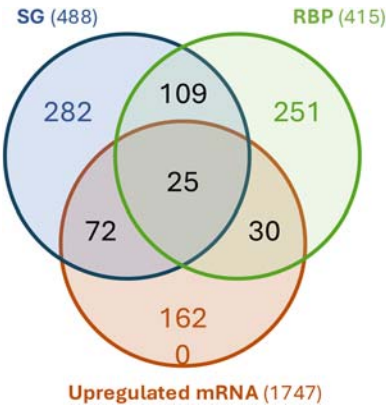

Figure EV1. Several mRNA coding for RBP and found in SG are present among the mRNA upregulated during pancreatic inflammation.

Venn diagram developed from transcriptomic data previously collected by our team (Assi et al, 2021), the RBP databank (http://rbpdb.ccbr.utoronto.ca/), and the SG databank (https://rnagranuledb.lunenfeld.ca/).

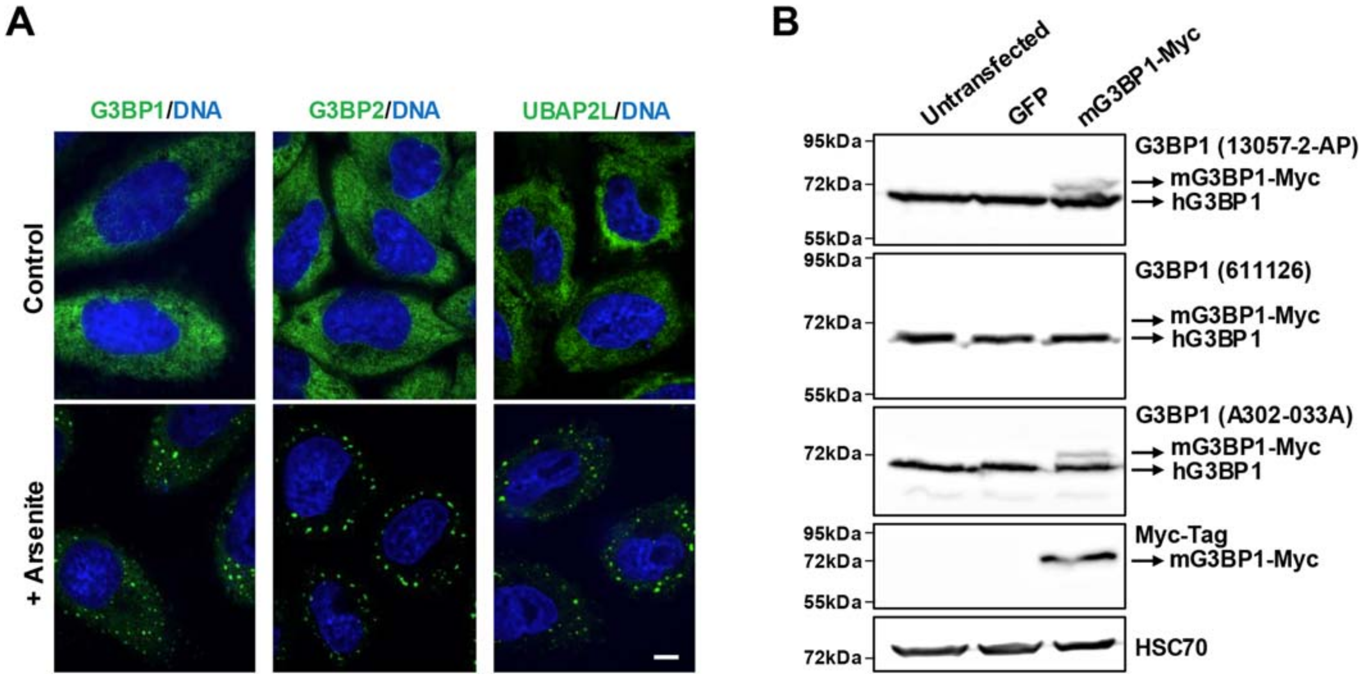

Figure EV2. Selected antibodies specifically recognize SG proteins.

(**A**) Immunolabelling performed with G3BP1 (13057-2-AP), G3BP2, and UBAP2L antibodies on untreated (top panels) or arsenite-treated (bottom panels) Hela cells. Scale bar: 10 μm. (**B**) Western blotting performed on protein extracts from untransfected, GFP-transfected, and mouse (m) G3BP1-transfected HeLa cells with G3BP1 (13057-2-AP), G3BP1 (611126), G3BP1 (A302-033A), and Myc-Tag antibodies. Human (h) G3BP1 was detected by the 3 G3BP1 antibodies, whereas mG3BP1 was recognized by G3BP1 (13057-2-AP) and G3BP1 (A302-033A) antibodies, but not by G3BP1 (611126) antibody. The pictures shown are representative of tissues from at least 3 biological replicates.

 © The Author(s)

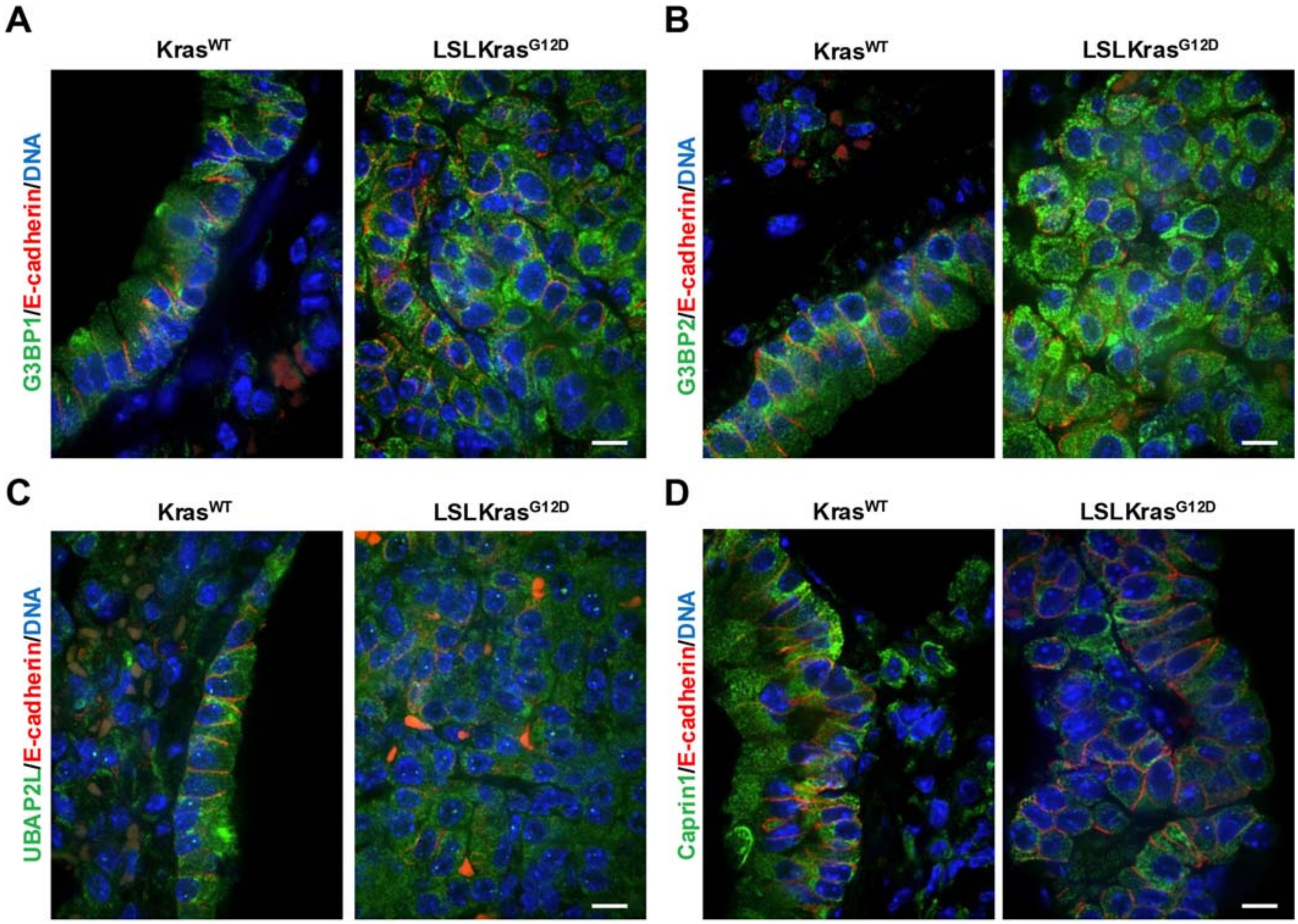

Figure EV3. SG are not observed in lung adenomas.

Immunolabeling performed with E-cadherin and G3BP1 (**A**), G3BP2 (**B**), UBAP2L (**C**), and Caprin1 (**D**) on lung sections of $Kras^{WT}$ and $LSLKras^{G12D}$ mice infected by intratracheal instillation with Cre adenovirus. Bronchial epithelium is shown for $Kras^{WT}$ lung sections while adenoma is exemplified for $LSLKras^{G12D}$ lung sections. Nuclei were counterstained with Hoechst. Scale bar: 10 µm. The pictures shown are representative of tissues from at least 3 biological replicates.

© The Author(s)
